# A Rare Paraspinal Desmoid Tumour following Instrumented Scoliosis Correction in an Adolescent

**DOI:** 10.1155/2021/6665330

**Published:** 2021-02-22

**Authors:** Fergus J. McCabe, Alan J. Hussey, John P. McCabe

**Affiliations:** ^1^Spine Service, Department of Trauma and Orthopaedic Surgery, Galway University Hospitals, Galway, Ireland H71 YR71; ^2^Department of Plastic and Reconstructive Surgery, Galway University Hospitals, Galway, Ireland H71 YR71

## Abstract

Desmoid tumours are benign neoplasms of myofibroblasts, often occurring after soft-tissue trauma. Rarely, desmoid tumours can occur following operative intervention, including spine surgery. In this case report, we describe the first reported case of desmoid tumour following scoliosis corrective surgery in an adolescent.

## 1. Introduction

Desmoid tumours, also known as 'aggressive fibromatosis' are rare, locally invasive but non-metastasising, neoplasms of fibroblasts or myofibroblasts [1]. First described by McFarlane in 1832 [2], but named “desmoid” by Müller in 1838 [3], the description derives from the Ancient Greek *desmos*, meaning 'band' or 'tendon-like' [4].

Desmoids are formed from the proliferation of myofibroblasts within connective tissue, resulting in a soft-tissue mass [5]. These tumours are most commonly found in the abdominal wall, mesentery, and limbs [1]. Desmoid tumours can invade surrounding tissues, often spreading along muscular planes. Females are twice more likely to be affected as men, primarily those of reproductive age [2, 4].

Although non-metastasising, desmoid tumours are particularly difficult to treat due to local invasion and post-resection recurrence of up to 50% [6].

Antecedent trauma in a well-recognised risk factor for desmoid tumours, present in up to 30% of cases [7]. Surgery, as a traumatic cause, has been described. There are 15 reported cases of paraspinal desmoid tumours following spine surgery [8–13].

Here, we describe the first reported case of a paraspinal desmoid tumour following scoliosis correction surgery in a minor.

## 2. Case Presentation

An otherwise healthy eight-year-old girl was referred to the spine service with a new onset spinal deformity. Radiographs revealed a 47-degree right-sided thoracic scoliosis consistent with juvenile idiopathic scoliosis. She had no family history of scoliosis.

The girl was fitted with a Boston-type brace and followed with serial imaging. The goal of treatment was to stabilise the curve to beyond the adolescent growth spurt.

**Figure 1:**
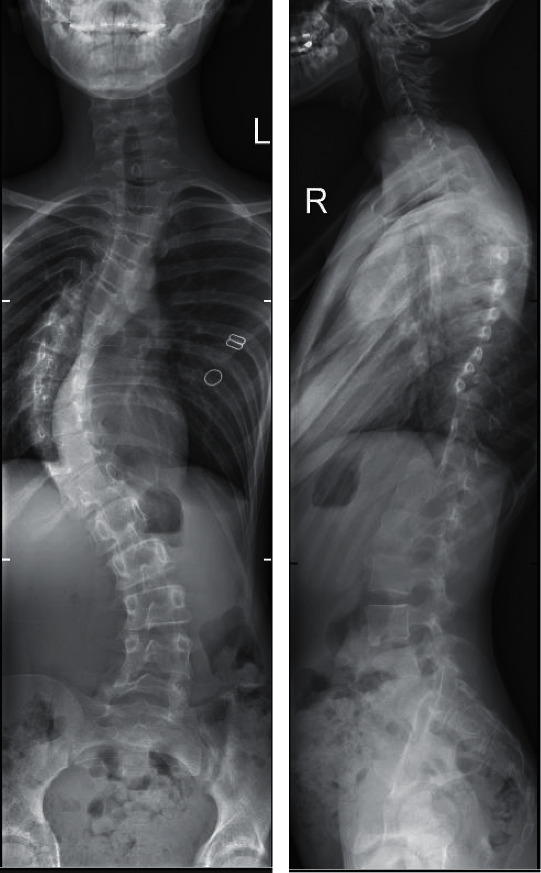
Anteroposterior and lateral X-rays demonstrating right-sided scoliosis.

At 13 years old, five years after initial presentation, the girl's curve had slowly progressed to 54 degrees (Figure 1). She opted for operative correction of her scoliosis.

**Figure 2:**
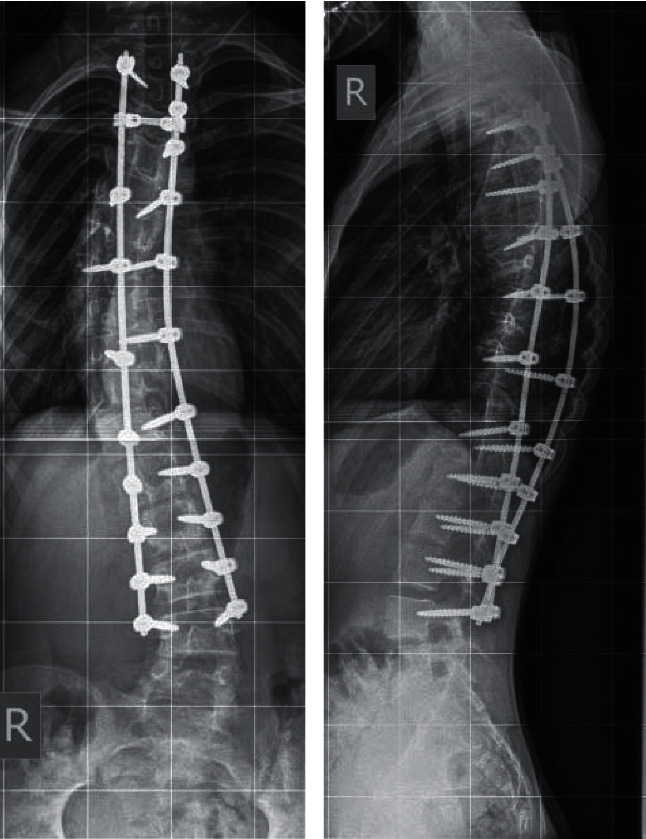
Anteroposterior and lateral X-rays demonstrating scoliosis correction with posterior instrumentation.

The girl underwent open posterior instrumented correction of T2 to L4 (Figure 2). She had an uneventful postoperative course and was discharged home five days later. Her wounds healed well and she returned to normal activity with time.

Three years later, now 16 years old, she developed a painful swelling on her upper right back. This increased in size over subsequent weeks, and she attended for review. On clinical examination, there was a tense, tender, right paraspinal mass, palpable from C7 to T2. She was admitted for urgent investigation.

**Figure 3:**
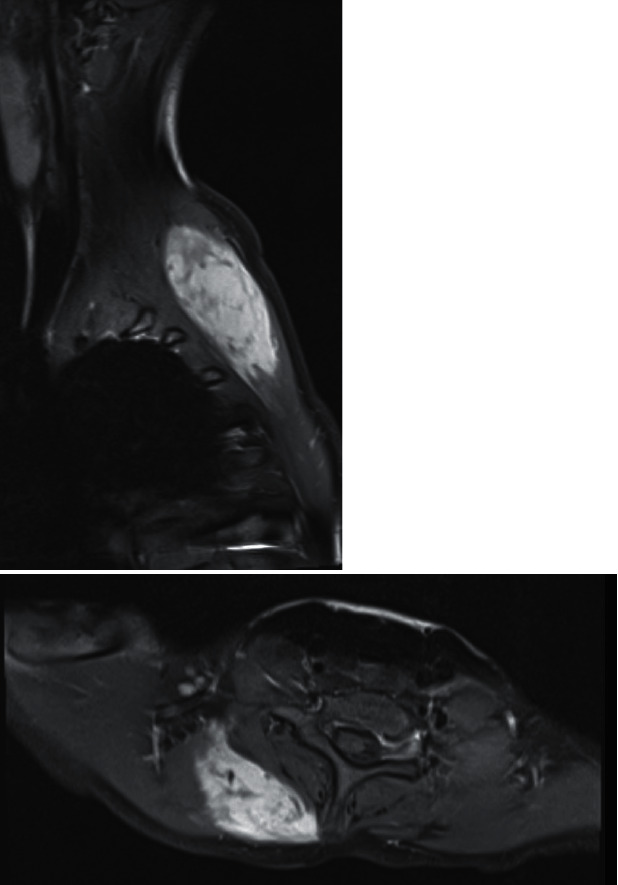
Right parasagittal and axial T1-weighted MRI sections demonstrating gadolinium contrast-enhancing right paraspinal mass consistent with a desmoid tumour.

MRI revealed a contrast-enhancing 3.3 cm x 7.0 cm x 8.4 cm right paraspinal mass, extending from C6 to T3 (Figure 3). The mass lay between the serratus anterior and trapezius but demonstrated no local invasion.

A subsequent biopsy of the mass was performed. The histopathology demonstrated a benign fibroblastic/myofibroblastic spindle cell neoplasm with predominant fascicular architecture. Mitoses were scarce, and there was no pleomorphism. Immunostains demonstrated multifocal positivity for smooth muscle actin (SMA) and multifocal nuclear positivity for beta-catenin. The appearances were most in keeping with an extra-abdominal desmoid tumour.

The case was discussed at multidisciplinary meeting. Given the high risk of recurrence with desmoid tumours and likelihood of incomplete resection, active surveillance was recommended unless increasing in size or highly symptomatic.

**Figure 4:**
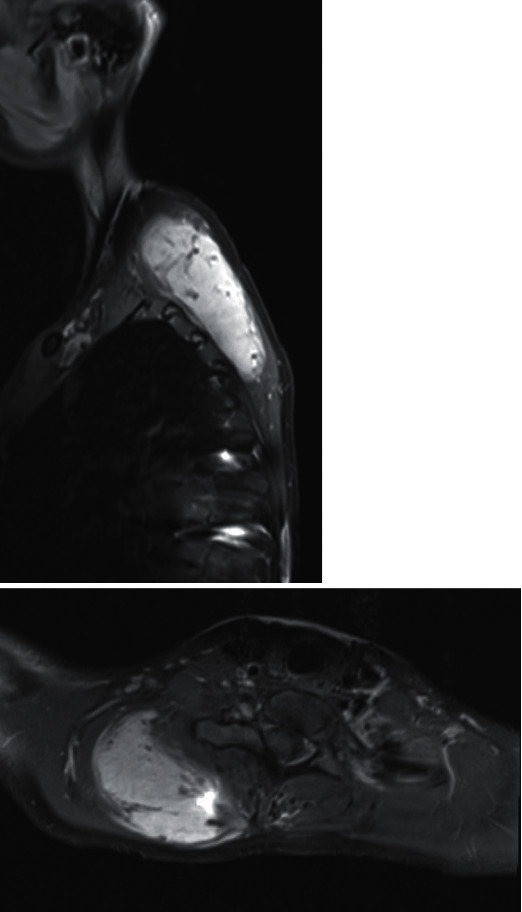
Right parasagittal and axial sections of T1-weighted MRI demonstrating increased size of contrast-enhancing paraspinal mass.

The young lady's tumour was monitored with serial MRI scans. After seven months, it had grown to 3.7 cm x 7.0 cm x 10 cm (Figure 4). Furthermore, her pain worsened considerably despite oral analgaesia. Uncontrolled tumour-related pain resulted in multiple missed school days and significantly limited the girl's quality of life.

With progression in size and symptoms, a multidisciplinary decision was made to proceed to operative resection. This was done under general anaesthetic via an extensile right upper thoracic incision extending towards her right scapula. The mass was excised en bloc. She had an uneventful postoperative course and was discharged well to home.

Histopathology revealed a 4.2 cm x 8.0 cm x 13.0 cm mass which once again confirmed desmoid tumour, without evidence of malignancy. Expert opinion was sought from Brigham and Women's Hospital, Boston, USA, which was accordant.

Although macroscopic margins were clear, there was one positive microscopic margin.

The girl was discharged well to home. Follow-up MRI showed no evidence of recurrence at 16 months.

## 3. Discussion

This is the first reported desmoid tumour following corrective surgery for juvenile idiopathic scoliosis. Furthermore, this is the youngest reported case of a post-operative paraspinal desmoid tumour.

Desmoid tumours account for 3% of soft-tissue tumours, and 0.03% of all neoplasms [14]. The fibromatoses are composed of monoclonal neoplastic proliferations of fibroblasts or myofibroblasts. The exact aetiology remains unclear.

A number of causative genes play a role. Mutations of beta-catenin has been implicated in the majority of sporadic desmoid tumours [15–17]. Beta-catenin, through the Wnt/*β*-catenin signalling pathway, is dysregulated in many cancer types, leading to modulation of the normal immune response to neoplasia [18].

The *apc* (adenomatous polyposis coli) gene, which regulates degradation of beta-catenin, is also implicated [19]. Familial adenomatous polyposis (FAP), the resulting syndrome of *apc* mutation, accounts for 10% of desmoid tumours [5]. Desmoid tumours are 1000 times more common in those with FAP [20]. Desmoid tumours affect 5 to 30% of FAP patients, especially those with the Gardner subtype [21].

Trauma is a recognised antecedent factor for a proportion of desmoid tumours [22]. One proposed hypothesis is that trauma, through inflammation, leads to upregulation of beta-catenin [20]. If beta-catenin degradation is dysregulated, the transcription factor can accumulate and upregulate *WISP1*, *SOX1*, *ADAM12*, and *Fap-1* resulting in cellular proliferation and production of extracellular matrix proteins [23]. Uncontrolled cellular proliferation results in the monoclonal fibroblast proliferation that is the hallmark of desmoids.

Spine surgery, as a cause of trauma, has been identified as a predisposing factor for desmoid tumour formation [8]. Postoperative paraspinal desmoids tend to occur in young females, though level of surgery or use of instrumentation does not appear to have an effect [8]. As mentioned, fourteen cases of post-spine surgery paraspinal desmoid tumours have been reported. This lady is the only reported case to occur following scoliosis corrective surgery in a minor.

Given the unpredictable clinical course, and possibility of spontaneous regression, appropriate management of desmoid tumours must be individualised [20].

Traditionally, the treatment of choice for desmoid tumours was wide resection with clear margins [2]. However, on account of their locally invasive nature, en bloc resection of these tumours can be highly morbid, while often failing to achieve microscopically clear margins [24]. Furthermore, even with negative margins, the risk of recurrence can remain high [6].

Recent evidence-based international consensus from the Desmoid Tumour Working Group advocates for active surveillance as the primary management strategy [24]. Any treatment decision-making should be done by a multidisciplinary team [24].

Where active treatment is required, to control invasion or for symptomatic relief, the Desmoid Tumour Working Group advocates surgery as first line, provided morbidity is limited [24]. The aim is a wide, microscopically negative resection margin (R0), but positive microscopic margins (R1) are acceptable in the event that a more extensive resection would compromise function or cosmesis [24].

While macroscopically positive margins convey greater risk of recurrence, there is growing evidence showing no difference in recurrence rates between microscopically positive resection and microscopically negative margins [6, 25].

There is no proven benefit of radiation therapy for microscopic positive margins; thus, it is no longer recommended [24]. Primary radiation or medical therapy are acceptable alternatives if surgical excision is likely to be morbid [24].

This young lady had a macroscopically negative, microscopically positive resection without adjuvant radiation therapy. The resection was limited to protect function of the adjacent musculature. She has no radiological evidence of recurrence at 16 months.

To assess the risk of recurrence following operative resection of desmoid tumours, Crago et al. developed a validated prediction tool using data from 439 patients [6]. They determined the major independent risk factors for recurrence to be age at diagnosis, size of tumour, and anatomical location [6]. Using this tool, the risk of recurrence for this young lady is 45% at 3 years, and 49% at 5 years [6].

## 4. Conclusion

This is the first reported case of desmoid tumour following juvenile idiopathic scoliosis corrective surgery. Furthermore, it is the youngest reported case of a desmoid following spine surgery. Active surveillance was initiated, but the tumour progressed on follow-up imaging. Operative resection was successfully performed. The risk of local recurrence remains.

## Data Availability

Patient data is confidential, thus will not be made publicly available.
